# A systematic review and meta-analysis of GPT-based differential diagnostic accuracy in radiological cases: 2023–2025

**DOI:** 10.3389/fradi.2025.1670517

**Published:** 2025-10-28

**Authors:** Daniel Nguyen, Isaac Bronson, Ryan Chen, Young H. Kim

**Affiliations:** ^1^ University of Massachusetts Chan Medical School, Worcester, MA, United States; ^2^ Department of Radiology, University of Massachusetts Chan Medical School, Worcester, MA, United States

**Keywords:** radiology, artificial intelligence, natural language processing, large language model, meta-analysis

## Abstract

**Objective:**

To systematically evaluate the diagnostic accuracy of various GPT models in radiology, focusing on differential diagnosis performance across textual and visual input modalities, model versions, and clinical contexts.

**Methods:**

A systematic review and meta-analysis were conducted using PubMed and SCOPUS databases on March 24, 2025, retrieving 639 articles. Studies were eligible if they evaluated GPT model diagnostic accuracy on radiology cases. Non-radiology applications, fine-tuned/custom models, board-style multiple-choice questions, or studies lacking accuracy data were excluded. After screening, 28 studies were included. Risk of bias was assessed using the Newcastle–Ottawa Scale (NOS). Diagnostic accuracy was assessed as top diagnosis accuracy (correct diagnosis listed first) and differential accuracy (correct diagnosis listed anywhere). Statistical analysis involved Mann–Whitney U tests using study-level median (median) accuracy with interquartile ranges (IQR), and a generalized linear mixed-effects model (GLMM) to evaluate predictors influencing model performance.

**Results:**

Analysis included 8,852 radiological cases across multiple radiology subspecialties. Differential accuracy varied significantly among GPT models, with newer models (GPT-4T: 72.00%, median 82.32%; GPT-4o: 57.23%, median 53.75%; GPT-4: 56.46%, median 56.65%) outperforming earlier versions (GPT-3.5: 37.87%, median 36.33%). Textual inputs demonstrated higher accuracy (GPT-4: 56.46%, median 58.23%) compared to visual inputs (GPT-4V: 42.32%, median 41.41%). The provision of clinical history was associated with improved diagnostic accuracy in the GLMM (OR = 1.27, *p* = .001), despite unadjusted medians showing lower performance when history was provided (61.74% vs. 52.28%). Private data (86.51%, median 94.00%) yielded higher accuracy than public data (47.62%, median 46.45%). Accuracy trends indicated improvement in newer models over time, while GPT-3.5's accuracy declined. GLMM results showed higher odds of accuracy for advanced models (OR = 1.84), and lower odds for visual inputs (OR = 0.29) and public datasets (OR = 0.34), while accuracy showed no significant trend over successive study years (*p* = 0.57). Egger's test found no significant publication bias, though considerable methodological heterogeneity was observed.

**Conclusion:**

This meta-analysis highlights significant variability in GPT model performance influenced by input modality, data source, and model version. High methodological heterogeneity across studies emphasizes the need for standardized protocols in future research, and readers should interpret pooled estimates and medians with this variability in mind.

## Introduction

Interest in artificial intelligence (AI) in radiology has grown in recent decades, with convolutional neural networks (CNNs) showing promise in tasks such as lesion detection, classification, segmentation, image reconstruction, and natural language processing (1). More recently, however, large language models (LLMs) like GPT (OpenAI, San Francisco, California) have garnered interest within radiology (2). LLMs are AI models that use transformer architectures to process and generate human language by learning from vast amounts of text data. These models understand the context of users’ queries, enabling them to perform various language-related tasks, including text generation, translation, and summarization. Unlike CNNs, which are trained on extensive labeled image datasets, GPT is trained on textual data using natural language processing. GPT-3.5 could generate differential diagnoses and management recommendations primarily from textual descriptions of imaging findings (3). However, GPT-4 and subsequent models are multimodal LLMs capable of processing text and images. This integration enhances their ability to generate comprehensive responses based on diverse inputs, for example, generating a differential diagnosis based on a patient history alongside either radiological images or transcribed interpretations of those images (3, 4). These GPT models, among more recently released versions such as GPT-o1 and GPT-o3-mini, are widely recognized LLMs that have demonstrated success in various medically related applications, such as clinical decision support and medical education (5, 6). Other LLMs, including Google's Gemini (Mountain View, California) and Anthropic's Claude (San Francisco, California), are also advancing rapidly. These models, along with GPT, all represent a growing body of tools with the potential to reshape healthcare through improved data analysis, language comprehension, and decision-making capabilities.

Researchers have published numerous examples of GPT's ability to generate differential diagnoses. For instance, a recently published study assessed GPT's ability to generate differential diagnoses from transcribed radiologic findings, revealing a diagnosis accuracy of 66.1% with GPT-4 (3). Another study evaluated GPT-4's performance using patient history and imaging findings from the “Diagnosis Please” quizzes in *Radiology*. The results showed a 54% accuracy in generating final diagnoses, with the highest accuracy in cardiovascular radiology (79%) and the lowest in musculoskeletal radiology (42%) (4). A separate study on thoracic radiology demonstrated that GPT-4 achieved a diagnostic accuracy of 59.7% when using complex, role-specific prompts, highlighting the importance of prompt engineering in optimizing model performance (7). Collectively, these findings suggest that while GPT has the potential to complement radiologic decision-making, its diagnostic accuracy and clinical reliability remain areas of active investigation. Further, the wide range in accuracy performance begs the question of what factors, such as prompt formulation, case complexity, imaging modality, and subspecialty, influence GPT's diagnostic performance.

Given these uncertainties, a comprehensive assessment of GPT's strengths, limitations, and areas for improvement is essential before considering its integration into radiologic workflows. In this meta-analysis, we compare the accuracy of various GPT models in generating differential diagnoses based on text and visual inputs of radiographic findings and patient histories to better characterize the strengths, weaknesses, and overall trajectory of GPT's diagnostic capabilities.

## Methods

Institutional Review Board (IRB) approval was not required because this meta-analysis used only previously published, de-identified data and did not involve human subjects or protected health information.

**Figure 1:**
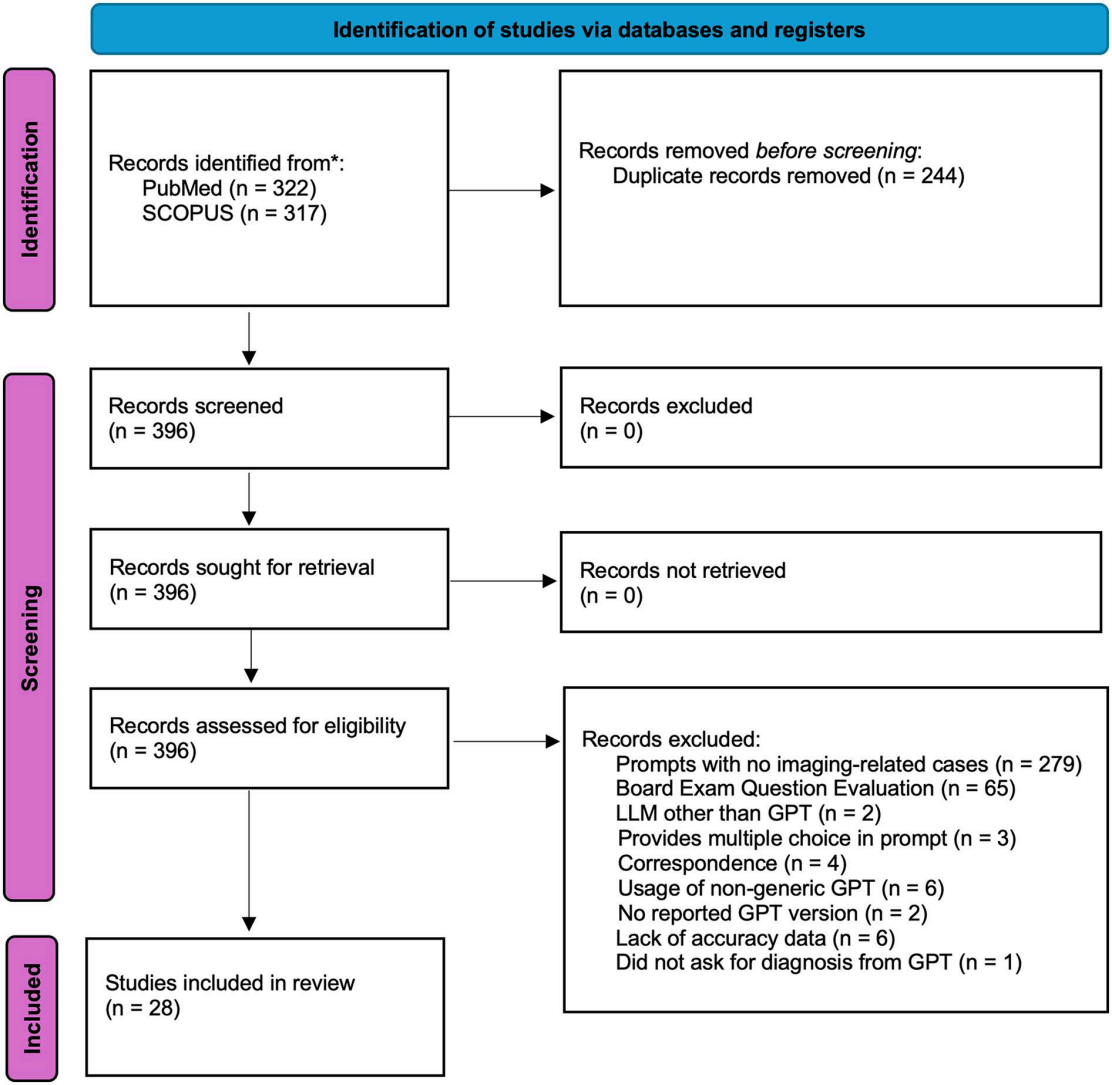
PRISMA diagram. The search strategy can be found in the Methods section of the manuscript. Records were retrieved from PubMed and SCOPUS. A total of *n* = 279 articles retrieved were found not to contain any imaging-related cases used for GPT diagnostic evaluation. A total of *n* = 65 studies evaluated GPT performance on board exam questions or questions that mimic board exam question style. A total of *n* = 6 studies also did not provide accuracy data that could be used for the subsequent statistical analysis completed in our meta-analysis.

This meta-analysis was not prospectively registered, and no formal review protocol was prepared. The study was conducted in accordance with PRISMA guidelines, and all inclusion/exclusion criteria, analysis plans, and outcomes were determined prior to data extraction. The PRISMA diagram of the workflow can be seen in Figure 1. 639 articles were retrieved from PubMed (*n* = 322) and SCOPUS (*n* = 317) on 3-24-2025. Search terminology for PubMed was “ChatGPT” OR “ChatGPT-4” OR “ChatGPT-3.5” OR “ChatGPT4” OR “ChatGPT4O” OR “ChatGPT-4o” OR “GPT” OR “Chat-GPT” OR “large language model” OR “artificial intelligence chatbots”) AND (“radiology” OR “radiologic”) AND (“accuracy” OR “diagnostic performance” OR “diagnose”). Search terminology for SCOPUS was (“ChatGPT” OR “ChatGPT-4” OR “ChatGPT-3.5” OR “ChatGPT4” OR “ChatGPT4O” OR “ChatGPT-4o” OR “GPT” OR “Chat-GPT” OR “large language model” OR “artificial intelligence chatbots”) AND (“radiology” OR “radiologic”) AND (“accuracy” OR “diagnostic performance” OR “diagnose”). Articles evaluating GPT performance on radiological cases with reported diagnostic accuracy were included. A total of *n* = 28 studies were included in this study after review by *n* = 2 research personnel with two years’ worth of research experience in the field of LLMs, followed by review by a board-certified radiologist (3, 4, 7–32).

### Eligibility criteria

Articles initially excluded from analysis were removed due to duplicates (*n* = 244), resulting in a total of 396 articles for retrieval. Following that, a total of *n* = 279 articles retrieved were papers that evaluated the accuracy of GPT in diagnosing non-imaging, non-radiology-related cases such as dermatological conditions, simulated clinical scenarios, or clinical vignettes. For example, this exclusion criterion included articles that provided a clinical vignette and asked GPT to manage medications, generate documentation, and/or guide the clinical management of a patient in a field unrelated to radiology. The remaining 117 articles were independently assessed for eligibility by two researchers.

Disagreements were resolved through discussion and consensus, or by involving third research personnel when consensus could not be reached. 279 retrieved articles were excluded based on the models being tested on basic trivia, general medical facts, and/or short questions related to radiology, which do not simulate real scenarios where GPT may be used in a clinical workflow. Similarly, 3 of the retrieved articles provided multiple-choice answers in the prompt in the form of either medical trivia/or board-style exam questions. Two of the retrieved articles used an LLM other than GPT. Other exclusion factors included correspondence (*n* = 4) and articles that did not report the GPT version used (*n* = 2). Interestingly, six articles used a version of GPT (non-generic GPT) that had been pre-trained with user-provided datasets. Studies evaluating fine-tuned GPT models were excluded to focus on publicly and commercially available versions. Finally, six retrieved articles did not report accuracy-level data that could be used in our meta-analysis. One of the retrieved articles did not explicitly ask GPT for a clinical diagnosis but instead asked to point out imaging findings (33).

### Data collection

Data extraction was conducted by *n* = 2 research personnel with two years’ worth of research experience in the field of LLMs. Data extracted from the *n* = 28 studies include the radiology topic being tested by the LLMs, whether patient history was provided in the context of the radiological case, GPT version, GPT access date, and whether the images or cases were obtained were publicly available on the internet or provided from a private source (such as institutional medical records). Accuracy was included and reported in two ways: top diagnosis accuracy, defined as GPT correctly listing the diagnosis as the first entry in its differential diagnosis, and differential accuracy, defined as GPT listing the correct diagnosis anywhere within its differential diagnosis. To identify a potential source of methodological variation, the size of differential diagnosis list (*k*) was extracted**.** As the *k* value could not be confirmed for 13 of the included studies, no formal moderator analysis was completed; however, the determined *k* values are noted in Table 1.

**Table 1:** Summary of literature and input types for each study.

Study name	Dataset	Textual input	Visual input	Grading scheme	*k* value
Brin et al. 2025 (31)	Acquired hospital images/reports	None	US, CT, XR	Differential	Undefined
Cesur et al. 2024 (7)	Thoracic Society of Radiology Case of the Month	Patient history, imaging findings	None	Differential	3
Dehdab et al. 2024 (30)	The Cancer Imaging Archive	None	CT	Differential	Undefined
Fink et al. 2025 (29)	Created written report findings	Imaging findings	None	Differential	Undefined
Hiredesai et al. 2024 (32)	Radiopaedia for 6 common upper extremity bony pathology	None	XR, CT, MRI	Differential	Undefined
Horiuichi et al. 2024 (26)	“Test yourself” cases from skeletal radiology	Patient history, imaging findings	Used, did not clarify which modalities were in the dataset	Both	3
Horiuichi et al. 2024 (27)	Neuropathology case conference cases from *Clinical Neuroradiology*	Patient history, imaging findings	Used, did not clarify which modalities were in the dataset	Both	Undefined
Horiuichi et al. 2023 (28)	*American Journal of Neuroradiology* Case of the Month	Patient history, imaging findings	None	Both	Undefined
Huppertz et al. 2025 (25)	Acquired hospital images/reports	Patient history	CT, MRI, Angiography	Both	Undefined
Kikuchi et al. 2024 (24)	*American Journal of Neuroradiology* Case of the Month	Patient history, imaging findings	None	Differential	3
Koyun et al. 2024 (22)	Acquired hospital images/reports	None	MRI	Differential	Undefined
Koyun et al. 2025 (23)	Acquired hospital images/reports	None	CT	Differential	Undefined
Li et al. 2024 (20)	Radiology Diagnosis Please archive	Patient history, imaging findings	None	Differential	5
Li et al. 2025 (21)	Radiology Diagnosis Please archive	Patient history, imaging findings	None	Differential	5
Mitsuyama et al. 2025 (19)	Acquired hospital images/reports	Imaging findings	None	Both	3
Mohammadi et al. 2024 (18)	Acquired hospital images/reports	None	XR	Differential	Undefined
Ozenbas et al. 2025 (17)	Acquired hospital images/reports	None	MRI	Both	3
Rau et al. 2024 (16)	Created written report findings	Patient history, imaging findings	None	Both	3
Reith et al. 2024 (15)	Publicly available images from Pediatric Imaging website	Patient history	XR, Fluoroscopy, US, CT, MRI	Differential	Undefined
Ren et al. 2024 (14)	Acquired hospital images/reports	None	XR	Both	3
Sonoda et al. 2024 (13)	Radiology Diagnosis Please archive	Patient history, imaging findings	None	Both	3
Sorin et al. 2024 (12)	Acquired hospital images/reports	Imaging findings	None	Differential	3
Strotzer et al. 2024 (11)	Acquired hospital images/reports	None	CT, MRI, XR	Both	5
Suh et al. 2024 (10)	Radiology Diagnosis Please archive	Patient history, imaging findings	XR, CT, US, MRI	Differential	3
Sun et al. 2024 (3)	Cases from “Top 3 Differentials in Radiology”	Imaging findings	None	Both	3
Suthar et al. 2023 (9)	*American Journal of Neuroradiology* Case of the Month	Patient history, imaging findings	None	Differential	Undefined
Ueda et al. 2023 (23)	Radiology Diagnosis Please archive	Patient history, imaging findings	None	Both	Undefined
Wada et al. 2024 (8)	*American Journal of Neuroradiology* Case of the Month	Patient history, imaging findings	None	Both	5

Below is a summary table of the *n* = 28 studies used in the meta-analysis. “Acquired hospital images/reports” signifies that that data (either the images themselves or the radiology report) was extracted from a hospital system and/or institution. Textual input is defined as additional, textual information that is added into the prompt. Visual input is defined as any image added into the prompt. Grading Scheme is defined as the grading system employed by the study, which is either differential accuracy (differential), top diagnosis accuracy (top), or both. The k value refers to the size of differential diagnosis list.

### Textual and visual inputs

**Figure 2:**
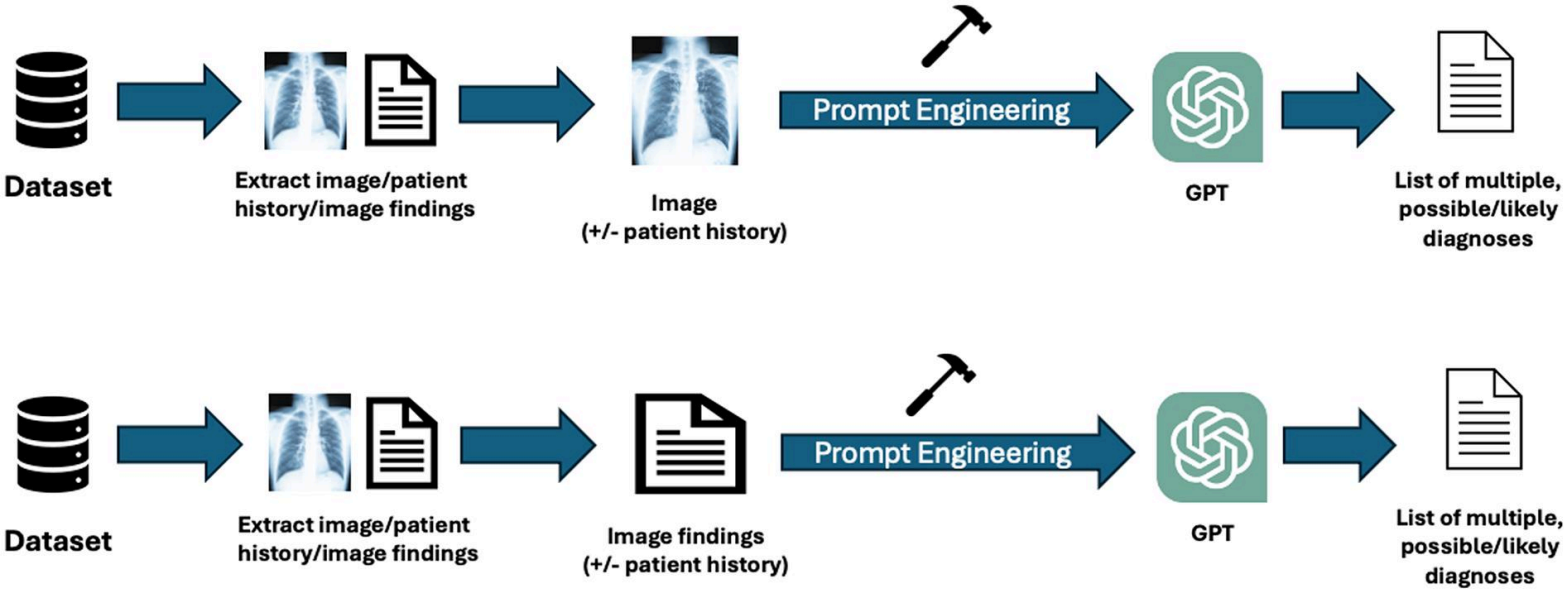
Workflow of inputs and outputs for GPT across the included studies: this figure illustrates the various methodologies used by visual or textual inputs across the studies. Researchers employed public or private datasets to extract selected images and relevant patient history or findings. Written image findings were used for textual inputs; the actual images were submitted for visual inputs. The inclusion of patient history varied by study. Prompt engineering was applied to format inputs according to individual study protocols or based on previously established literature. The outputs generated by GPT models were either a list of potential diagnoses or a single most likely diagnosis.

Among the *n* = 28 studies included, the types of textual and visual inputs provided to GPT models varied widely depending on study design. Textual inputs primarily consisted of descriptions of imaging findings, sourced either from radiology reports within de-identified institutional datasets or from online resources such as the American Journal of Neuroradiology Case of the Month series or Radiology: Diagnosis Please cases. Several studies evaluated the impact of providing clinical context, such as age, symptoms, or referring physician notes. In contrast, others tested the model's ability to generate interpretations based solely on imaging descriptions without supplementary history. Visual inputs, when used, included static images (e.g., x-rays [XR], CT scans [CT], magnetic resonance imaging [MRI], or ultrasound [US] screenshots), either embedded directly into the prompting interface (for multimodal-capable models) or described textually in image captions. A summary of the inputs can be found in Table 1. The different methodologies by which the inputs were utilized can be found in Figure 2.

### Bias assessment

We assessed risk of bias for each included study using the Newcastle-Ottawa Scale (NOS), focusing on selection, comparability, and outcome domains. This scale has been previously used in a meta-analysis that assessed GPT accuracy in answering medical queries, which we had adapted to this meta-analysis in assessing GPT diagnostics (34). Two independent reviewers assessed all studies based on the NOS framework, which evaluates risk of bias across three core domains: (1) selection of study cohorts or designs, (2) comparability of groups, and (3) ascertainment of outcomes. For domains D1-D4 (Supplementary Figure S1), studies were judged on criteria such as the clarity and representativeness of included cases. The comparability domain D5-D6 (Supplementary Figure S1) assessed how well studies controlled for potential confounders, such as model version or input modality. The outcome domain D7-D9 (Supplementary Figure S1) is the appropriateness of outcome measurement. Each criterion (D1-D9) was scored as follows: none (0 points), unclear (0.5 points), and yes (1 point). The results from both reviewers were summed to produce an overall score for each study (maximum total = 18). Based on the total NOS scores, studies were categorized into high (dark red, 0–6 points), moderate (dark yellow, 7–12 points), or low risk of bias (dark green, 13–18 points). The risk of bias ratings was factored into our interpretation of pooled results and heterogeneity.

### Statistical analysis

Data from the included radiology studies were analyzed using R (version 4.2.2). Descriptive statistics were used to summarize the evaluated radiologic topics and the GPT models’ diagnostic performance across tasks. Accuracy was calculated for both top diagnosis (correct diagnosis listed first when GPT provides a differential diagnosis) and differential diagnosis (correct diagnosis listed anywhere in the list), reported as proportions with 95% confidence intervals (CI) derived from standard errors under binomial distribution assumptions. The first scheme is defined as top diagnosis accuracy, and the second is defined as differential accuracy. Across the *n* = 28 studies, a correct diagnosis is defined as a diagnosis that matches the ground truth. In studies using publicly available cases (e.g., the American Journal of Neuroradiology or Radiology: Diagnosis Please), the correct diagnosis was typically defined as the official answer provided by the source. In studies using de-identified institutional datasets, the ground truth was defined as the attending radiologist's final diagnosis documented in the official radiology report. This meta-analysis relied on each study's reported accuracy values and did not re-evaluate model predictions independently.

To account for clustering of multiple cases within each study, we collapsed results to the study level by summing the number of correct predictions and total cases for each article, stratified by GPT model, input modality, history provision, and data source. From these counts, we calculated study-level accuracy proportions (both top diagnosis and differential diagnosis). For descriptive reporting, we summarized pooled accuracy as well as the study-level median accuracy and interquartile range (IQR) across included studies. Between-group comparisons (e.g., GPT-3.5 vs. GPT-4 family models, textual vs. visual inputs, history provision, and public vs. private datasets) were assessed using two-sided Mann–Whitney U tests (Wilcoxon rank-sum), which compare distributions without assuming normality.

To evaluate predictors of diagnostic accuracy while adjusting for potential confounders, we constructed a generalized linear mixed-effects model (GLMM). The dependent variable was correct vs. incorrect diagnosis at the case level, expanded from study-level counts. Fixed effects included GPT model type (GPT-4 family vs. GPT-3.5), input modality (textual vs. visual), history provision (yes vs. no), data source (public vs. private), and study year (continuous). A random intercept for each study was included to account for within-study clustering. Model results were expressed as odds ratios (OR) with 95% confidence intervals, where OR > 1 indicates higher odds of accuracy.

A funnel plot was generated using a fixed-effect model to assess publication bias, and Egger's regression test was applied. All statistical tests were two-sided, with significance defined as *p* < 0.05.

## Results

The search strategy yielded 639 records from PubMed (*n* = 322) and SCOPUS (*n* = 317). After removing duplicates (*n* = 244), 396 articles remained for screening. Following a detailed assessment for eligibility, 28 studies were included in the final analysis (Figure 1; Table 1).

The included studies evaluated various radiology subspecialties, most frequently neuroradiology (60.71%), musculoskeletal radiology (42.86%), and chest radiology (25.00%). Other notable areas included breast imaging (17.86%), gastrointestinal imaging (21.43%), cardiovascular imaging (17.86%), genitourinary imaging (17.86%), and pediatric radiology (14.29%). We found that several studies (14.29%) did not specify the radiology subspecialty tested (Table 2). A total of *n* = 8,852 radiological cases (whether the images themselves or the radiological image described in text) were evaluated across all the included *n* = 28 studies. In the meta-analysis data, GPT-4 was the most tested GPT model with *n* = 2,662 radiological cases, followed by GPT-4V with *n* = 2,306 radiological cases (Table 3).

**Table 2:** Stratification of radiology topics tested.

Tested radiology topics	*N*	Frequency (%)
Neuroradiology	17	60.71
Musculoskeletal	12	42.86
Chest	7	25.00
Gastrointestinal	6	21.43
Breast	5	17.86
Cardiovascular	5	17.86
Genitourinary	5	17.86
Pediatric	4	14.29
Not reported	4	14.29
Head and neck	3	10.71
OB/GYN	3	10.71

A table showing a list of all the different radiology topics that were reported in review of all published papers. This table only displays radiology topics more than *n* = 2 times. Also displays the frequency which is that number divided by *n* = 28 total studies. Not Reported signifies the number of studies that did not report the radiology topic.

**Table 3:** Average accuracy table of GPT-4, GPT-4o, and overall for Top diagnosis vs. differential diagnosis.

GPT model	Top accuracy			Differential accuracy		
	Accuracy [95% C.I]	Study-level median accuracy	IQR	Accuracy [95% C.I]	Study-level median accuracy	IQR
GPT-3.5	150/293 (51.21%) [45.47, 56.91]	55.92%	0.00	636/1680 (37.87%) [35.53, 40.17]	36.33%	12.62
GPT-4	614/1075 (57.1%) [55.63, 63.01]	57.82%	50.82	1514/2662 (56.46%) [54.57, 58.34]	**56.65%十**	10.58
GPT-4T	414/751 (55.12%) [51.56, 58.58]	55.21%	27.69	577/801 (72.00%) [68.95, 73.59]	**82.32%十**	11.72
GPT-4V	343/1023 (33.53%) [30.63, 36.42]	33.33%	20.88	976/2306 (42.32%) [40.30, 44.34]	41.41%	30.97
GPT-4o	146/368 (39.67%) [34.68, 44.67]	35.35%	14.81	803/1403 (57.23%) [54.64, 59.82]	**53.75%十**	14.52
Overall	1667/3510 (47.50%) [45.84, 49.18]	48.36%		4506/8852 (50.92%) [49.95, 51.47]	47.21%	

This table presents the average accuracy of GPT-3.5, GPT-4, GPT-4V, GPT-4T, GPT-4o, and overall performance for top diagnosis vs. differential diagnosis. Accuracy is calculated as the number of correct cases divided by the total number of cases used, alongside the 95% confidence interval in brackets. Results were collapsed to the study level by summing correct predictions and total images per study. For each subgroup, we report the Study-level median accuracy and the interquartile range (IQR; Q3–Q1). A Mann–Whitney test is also reported to compare accuracy (either Top Accuracy or Differential Accuracy) performance between GPT-3.5 vs. the other GPT models, which displays significance at *p* < .05 (**十**).

Bold indicates statistical significance.

### Model performance comparison

The accuracy of GPT models varied significantly, especially in the context of the GPT model being employed. A total of *n* = 13 studies provided the top diagnosis accuracy, whereas all *n* = 28 studies provided differential accuracy. GPT-4 outperformed GPT-3.5 in both top diagnosis (57.1% vs. 51.1%) and differential accuracy (56.5% vs. 37.9%) at a statistically significant level. Accuracy was even higher for GPT-4T (72.0%) and GPT-4o (57.2%) for differential accuracy (Table 3). The overall differential accuracy across all GPT models was 50.92% (study-level median accuracy: 47.21%) (Table 3). Because all the *n* = 28 studies provided differential accuracy, this metric of accuracy was used as the accuracy metric for subsequent analysis.

### Textual vs. visual analysis

Comparing textual and visual modalities, GPT-4T achieved 94% accuracy with textual input but only 67% with visual input, based on a single study. GPT-4o performed slightly better with visual (59.6%) than textual (49.4%) inputs, though not significantly. GPT-4's textual accuracy was 56.5%, and GPT-3.5 reached 37.9%; visual data were unavailable for these models (Table 4).

**Table 4:** Comparison of textual GPT versus vs. GPT differential accuracy.

GPT functionality	Total cases	Accuracy [95% C.I]	Study-level median accuracy	IQR
GPT-3.5				
Textual	1,680	37.85% [35.45, 40.17]	35.40%	12.63
Visual	NA	NA	NA	NA
GPT-4				
Textual	2,662	56.46% [54.57, 58.34]	58.23%	10.52
Visual	NA	NA	NA	NA
GPT-4T				
Textual	50	94.00% [87.45, 100.00]	94.00%	0.00
Visual	751	66.97% [63.61, 70.34]	70.60%	0.00
GPT-4V				
Textual	NA	NA	NA	NA
Visual	2,306	42.32% [40.30, 44.34]	38.95%	30.92
GPT-4o				
Textual	324	49.38% [43.93, 54.82]	49.40%	0.00
Visual	1,079	59.59% [56.66, 62.59]	56.30%	16.46
Textual vs.visual	Total cases	Accuracy	Study-level median accuracy	IQR
Textual				
(−) Patient history	1,408	49.12% [46.60, 51.83]	**61.74%十**	47.81
(+) Patient history	3,308	49.96% [48.26, 51.67]	52.28%	17.69
Visual				
(−) Patient history	1,941	53.60% [51.42, 55.83]	54.29	30.28
(+) Patient history	2,195	49.29% [47.20, 51.38]	42.59%	30.77
Data acquisition	Total cases	Accuracy	Study-level median accuracy	IQR
Textual				
Private data	260	86.51% [82.42, 90.7]	**94.00%十**	16.23
Public data	4,456	47.62% [46.19, 49.12]	46.45%	25.58
Visual				
Private data	1,863	51.03% [48.83, 53.15]	52.25%	79.90
Public data	2,273	52.73% [50.79, 54.82]	46.73%	23.28

This table compares the performance of Textual GPT vs. Visual GPT on Differential Accuracy, for each model. GPT vs. Visual GPT on Differential Accuracy when provided with patient history is also compared on this table. This table compares the performance of GPT when using private vs. public data feeds. Public data refers to datasets that are openly available, whereas private data consists of information that is not accessible to the public such as confidential patient data. Results were collapsed to the study level by summing correct predictions and total images per study. For each subgroup, we report the Study-level median accuracy and the interquartile range (IQR; Q3–Q1). Group comparisons were performed using two-sided Mann–Whitney *U* tests (Wilcoxon rank-sum), which assess differences in distributions without assuming normality. Significance is denoted at *p* < .05 (**十**).

Bold indicates statistical significance.

### Impact of patient history provision

Among the studies, some included pertinent patient clinical information for the radiological case in the user-inputted prompt of GPT, whereas others did not. As such, we sought to tabulate the impact this provision had on the GPT differential accuracy. Providing patient history for textual inputs reduced overall accuracy (from 61.7% median to 52.3% median, *p* < .05). For visual inputs, accuracy also declined (from 54.3% to 42.6%), although not at statistical significance (Table 4).

### Public vs. private data sources

It is well known that GPT models are trained based on information provided on the Internet, with some models even having access to the Internet, such as GPT-4o. During this analysis, we found that differential accuracy differed by data source. For textual inputs, private datasets achieved much higher accuracy (86.5%) than public datasets (47.6%, *p* < .05). No significant differences were seen for visual inputs (private 51.0% vs. public 52.7%) (Table 4).

### Accuracy trends over time

GPT performance demonstrated variability over the years. Notably, GPT-4 accuracy increased from 57.49% in 2023 to 70.91% in 2025, while GPT-3.5 showed declining performance over time from 38.77% in 2024 to 26.10% in 2025. GPT-4T achieved a high accuracy of 94% in 2025, marking an improvement from 66.97% in 2024 (Supplementary Table S2), although this was just reported for one study.

### Generalized linear mixed-effect model analysis

We conducted a generalized linear mixed-effect model (GLMM) to identify key diagnostic accuracy predictors and assess how variables such as model version, input modality, data source, and study year influenced performance outcomes. More advanced GPT versions (GPT-4T/4 V/4/4o) were more likely (OR = 1.84, *p* < .001) to provide accurate diagnoses compared to GPT-3.5. Visual analysis had lower accuracy odds than textual analysis (OR = 0.29, *p* < .001). Public data sources had significantly lower odds of accuracy than private data (OR = 0.34, *p* < .001). Cases with patient history provided yielded a higher differential accuracy compared to those without (OR = 1.27, *p* = .001). There was no statistically significant difference over successive study years (OR = 1.19 per year, *p* < .001) (Table 5). We also removed *n* = 3 studies that were noted to have a moderate risk of bias as demonstrated in Supplementary Figure S1 (14, 23, 30), where we found that the results remained unchanged in direction or significance (Supplementary Table S2).

**Table 5:** Generalized linear mixed-effects model (GLMM) results of GPT differential accuracy across Key variables.

Predictor variable	Odds ratio (SE)	95% CI	*P* value
Differential diagnosis accuracy			
GPT-4T/4V/4/4o (vs. GPT-3.5)	1.84	(1.61, 2.12)	<.**001**
Visual analysis (vs. Textual analysis)	0.29	(0.19, 0.44)	**<**.**001**
Provided history: Yes (vs. No)	1.27	(1.10, 1.47)	**<**.**001**
Data acquisition: public (vs. Private)	0.34	(0.17, 0.70)	**<**.**001**
Year (continuous, 2023−2025)	1.19	(0.65, 2.20)	.57

Results of the generalized linear mixed-effects model (binomial, random intercept by study) evaluating predictors of ChatGPT differential diagnostic accuracy across included studies (2023–2025). Odds ratios greater than 1 indicate improved accuracy, while values less than 1 indicate reduced accuracy. Significant predictors included ChatGPT model type, modality (text vs visual), history provision, and data source. The fixed effects are the predictors, whereas the random effect is the study-level intercept, which accounts for clustering of multiple observations within the same article.

Bold indicates statistical significance.

### Heterogeneity assessment

**Figure 3:**
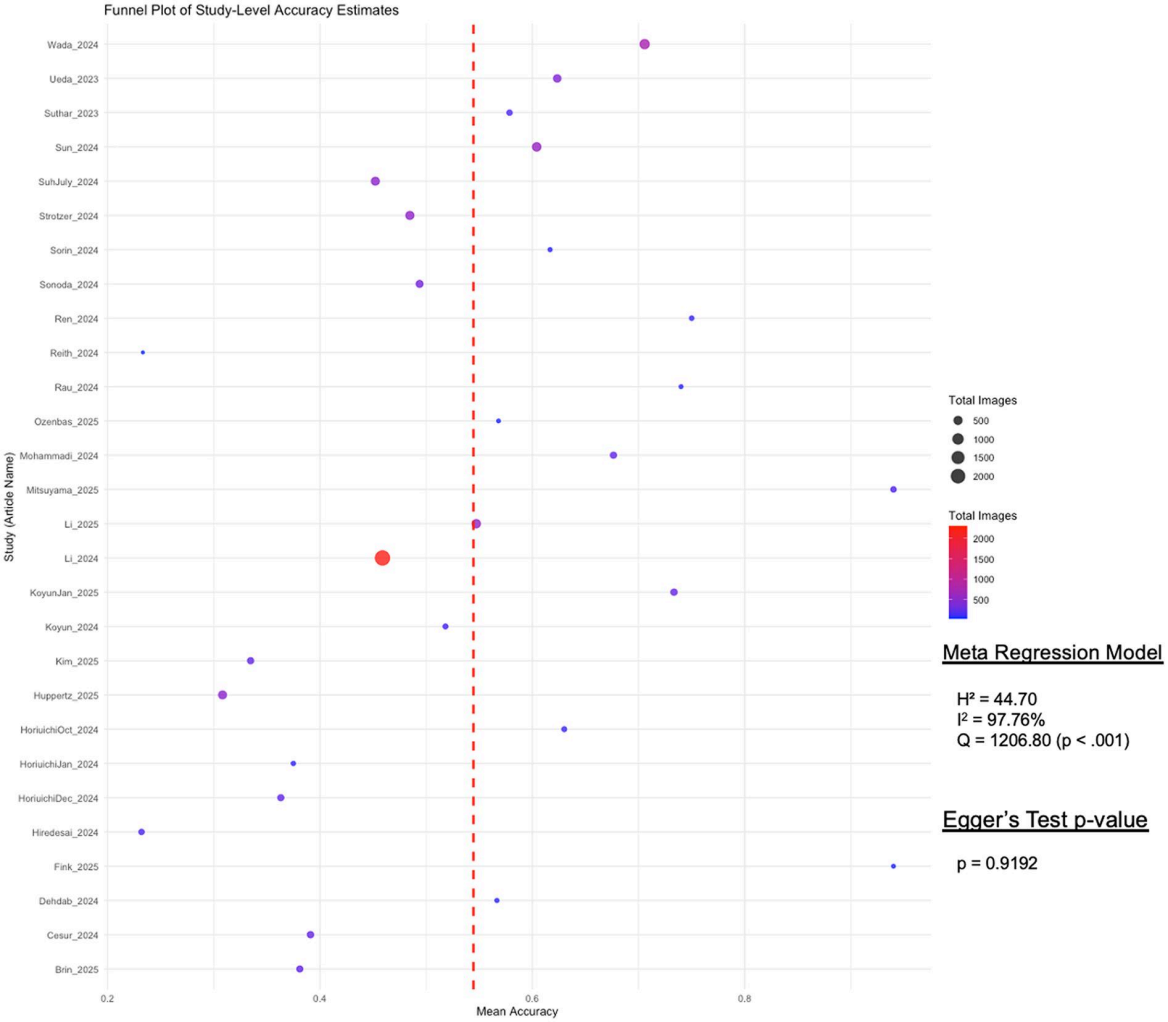
Funnel plot of study-level differential accuracy estimates (fixed-effects model) with Egger's test for publication bias. This funnel plot visualizes study-level accuracy estimates for GPT's differential accuracy. Each point represents a study, with mean accuracy on the *x*-axis and study names on the *y*-axis. Point size and color indicate the total number of images used per study, with a red dashed line marking the overall mean accuracy. Egger's test assesses publication bias, displaying its *p*-value to determine statistical significance.

To assess the presence of publication bias, we conducted a funnel plot visualization and applied Egger's regression test. The funnel plot (Figure 3) visualized study-level differential accuracy estimates, plotting mean accuracy along the *x*-axis and individual studies along the *y*-axis. The Fixed-Effects Model with *n* = 28 studies resulted in an average accuracy of 54.75% (95% CI: 53.78, 55.72%). While moderate heterogeneity was observed across studies (Q = 1206.80, *p* < 0.001; *I*^2^ = 97.76%), Egger's regression test revealed no statistically significant asymmetry (*p* = 0.9192), suggesting no substantial publication bias in the included literature.

## Discussion

In this meta-analysis, we provided essential insights into the diagnostic capabilities of various GPT models when applied to radiological cases. Newer GPT models demonstrated significantly higher diagnostic performance compared to earlier versions. This improvement aligns with advancements in multimodal capabilities, suggesting that continual refinement of transformer-based architectures significantly boosts their clinical utility (35). This pattern indicates that future iterations of LLMs may become increasingly reliable adjuncts to radiologists, particularly if their accuracy continues to evolve at a similar pace. However, caution is warranted as our analysis revealed widespread fluctuations in performance that vary between studies.

Although not statistically significant, the analysis revealed modality-specific strengths and weaknesses. GPT-4T excelled in textual inputs, achieving exceptionally high accuracy, whereas its performance notably declined with visual inputs. However, this result was based on only *n* = 50 cases from a single study. With limited sample sizes and potential selection bias, these cases may not represent the full scope of radiology diagnostics. On the other hand, GPT-4o demonstrated stronger diagnostic accuracy from visual inputs compared to textual descriptions. Previous studies have shown consistently poorer visual input performance than textual inputs (4, 36, 37). The observed discrepancy in GPT-4o's visual performance compared to the literature may be influenced by the broad methodological variability across studies included in this meta-analysis. Factors such as differences in data sources (public vs. private), year of model use, and variability in prompt engineering likely confounded these results, emphasizing the need for standardized methodologies in future investigations. Nonetheless, our GLMM results revealed that visual inputs had lower odds of accuracy than textual inputs. This difference in performance might reflect ongoing challenges with visual recognition and interpretation, as evidenced by the literature (2, 36). The observed distinction in performance may be attributed to the fundamental differences in how LLMs process these modalities. Textual prompts are handled via token-based transformer architectures optimized for language inputs, whereas visual inputs rely on separate encoders (38). In current models, this vision-language integration remains limited. Unlike textual prompts, which often already provide structured summaries of the imaging findings, visual prompts require the model to extract raw spatial features, for which GPT may not be optimized.

GPT-based models in radiological applications exhibited several distinct categories, or “types” of mistakes. One of the most prevalent issues reported in the literature is hallucination, where the model fabricates findings or diagnoses without any basis in the actual image data. For instance, GPT-4V has been shown to generate diagnoses and conclusions unsupported by the underlying images (25, 31). Even when prompted to provide up to five differential diagnoses, GPT-4T demonstrated a 29.4% misdiagnosis rate (221/751 cases), where the correct diagnosis was not included in its top five suggestions (8). Another study highlighted that while GPT models could detect the presence of intracranial hemorrhages, they struggled to accurately classify the hemorrhage type or localize them within the brain (23). The errors observed in GPT-based models underscore the critical need for further research, particularly given the potential risks that hallucinations and diagnostic inaccuracies pose if such systems are integrated into clinical practice.

Despite the absence of significant publication bias detected by Egger's test, our analysis revealed heterogeneity across included studies, reflecting considerable differences in methodologies, radiological cases, diagnostic criteria, prompt engineering approaches, and evaluation standards. The heterogeneity likely reflects the influence of additional unmeasured factors, most notably variations in prompt design. Prior studies have shown that prompt engineering can significantly enhance GPT's diagnostic performance (39). However, the included studies in our meta-analysis employed at least 10 distinct and non-overlapping prompting strategies (Table 6), making their inclusion as covariates difficult due to concerns of model overfitting. This methodological diversity significantly impacts the ability to generalize findings and directly compare study results. Such variability highlights the critical need for standardized evaluation protocols in future research.

**Table 6:** Examples of different prompting strategies used across selected studies (*n* = 28).

Study name	Strategy name	Prompting example
Cesur et al. 2024 (7)	“Physician”	"As a physician, I plan to utilize you for research purposes. Assuming you are a hypothetical physician, please walk me through the process from differential diagnosis to the most likely disease step by step, based on the patient's information I am about to present. Please list three possible differential diagnoses in order of likelihood."
	“Task”	''Give the most likely diagnosis and provide three differential diagnoses for each case below.''
	“Specific task”	''Your task is to analyze patient histories and imaging findings to give the most likely diagnosis and provide three differential diagnoses for each case below.''
	“Specific role”	''As a highly experienced Professor of Radiology with 30 years of expertise in thoracic imaging, you assist in solving thoracic radiology cases. Your task is to analyze patient histories and imaging findings to give the most likely diagnosis and provide three differential diagnoses for each case below.''
	“Exemplar”	''As a highly experienced Professor of Radiology with 30 years of expertise in thoracic imaging, you assist in solving thoracic radiology cases. Your task is to analyze patient histories and imaging findings to give the most likely diagnosis and provide three differential diagnoses for each case below. To complete this task, review the patient history and imaging findings provided for each case, analyze the data thoroughly, utilize your extensive knowledge in thoracic imaging, ensure that your diagnoses are well-supported, and make thoughtful decisions.''
Horiuchi et al. 2024 (26)	Chain-of-thought	“As a physician, I plan to utilize you for research purposes. Assuming you are a hypothetical physician, please walk me through the process from differential diagnosis to the most likely disease step by step, based on the patients information I am about to present. Please list three possible differential diagnoses in order of likelihood”
Mitsuyama et al. 2025 (19)	Simple	“List three possible differential diagnoses in order of likelihood from the following head MRI findings.”
Ozenbas et al. 2025 (17)	Guided chain-of-thought	"Which MRI sequences are present in the image?” “In which lobe or localization of the brain is the lesion located?” “What are the MRI signal characteristics of the lesion?” “Is there edema around the lesion?” “Is the lesion enhancing?” “Is the lesion intra-axial or extra-axial?” What are the three most likely differential diagnoses for the lesion?” “What is the most likely diagnosis for the lesion?"
Sun et al. 2024 (3)	Rationale	“What is the top 3 differential diagnosis for this scenario? Please explain why and provide your citations in standard AMA format. What is the most likely diagnosis for this scenario? Please explain why and provide your citations in standard AMA format.”
Wada et al. 2024 (8)	Role-playing, chain of thought, confidence assessment	# Role You are an expert in medical imaging diagnosis with extensive experience interpreting various medical images, including CT, MRI, and x-rays. Your expertise includes identifying pathologies, understanding radiology clinical report contexts, and correlating to imaging findings with potential diagnoses proofread. —# Request Along with the following Regulation prompt, present a refined list of five differential diagnoses, including the most probable diagnosis and four alternatives. Each diagnosis should have a corresponding confidence level based on your comprehensive analysis. —# Regulation Using the clinical information provided: {# URL of clinical information}, list five initial differential diagnoses. Then, review the imaging findings: {# URL of image findings}, and update your diagnoses accordingly. Reflect on how the new data alters your assessment. For each diagnosis in your updated list, assign a confidence level between 0% and 100%, considering the task's complexity and the extent to which clinical and imaging data support each diagnosis.

Given the heterogeneity of prompting methods among the reviewed studies, this table highlights select strategies employed by the authors. Strategy names enclosed in quotations (“ “) represent the original terminology used by each respective study. “Chain-of-Thought” refers to prompting GPT to explicitly explain each reasoning step; “Guided Chain-of-Thought” involves prompting GPT through a sequence of specific guiding questions; “Rationale” refers to requests for GPT to provide justifications for its answers; and “Confidence Assessment” describes prompts asking GPT to assign confidence levels to its responses.

Interestingly, unadjusted subgroup analyses suggested lower study-level median accuracy when clinical history was provided in textual prompts. However, in our multivariable GLMM, which accounted for confounding factors such as model version, data source, and modality, history provision was associated with significantly improved odds of higher differential accuracy. This apparent discrepancy may reflect confounding observed in the unadjusted subgroup analysis, as studies providing history may have used challenging cases or public datasets, which independently reduced performance. Previous studies have shown that providing additional information, such as in the form of prompt engineering, has been shown to improve the accuracy of GPT (39, 40). But in some cases, the added history can even mislead the AI, as evidenced by a larger drop in GPT's accuracy when the history contained distracting, biasing information (41). Taken together, our results confirm that clinical history provision does improve the performance of GPT, but should be interpreted with caution, given the methodological heterogeneity of the studies in this analysis.

Another intriguing observation was the higher accuracy obtained from private datasets than from publicly sourced data, especially for textual inputs. This could indicate that publicly available data, which often lacks detailed contextual information, might not provide sufficient depth for accurate model training or inference. Additionally, the superior accuracy of private datasets emphasizes the potential benefits of training or fine-tuning LLMs on institution-specific data to enhance clinical applicability, although privacy considerations remain crucial.

Moreover, the temporal analysis revealed distinct trends based on model version. We observed the improved performance of GPT-4 models over time, in contrast to the declining accuracy of GPT-3.5. This divergence likely reflects the evolving training methods and improved architecture of newer models, better equipped to handle increasingly complex medical tasks. Despite model-specific trends, our GLMM indicated that the continuous effect of the study year was not statistically significant. Together, these findings may suggest that performance trajectory is primarily dictated by architectural advancements rather than external temporal factors. It also highlights the need for ongoing evaluation of GPT performance, particularly as newer versions are introduced and tested in increasingly complex clinical scenarios.

Limitations of this meta-analysis include variability in study design, methods of reporting accuracy, and heterogeneity in the datasets used. Different prompt engineering techniques and a lack of standardized evaluation criteria across studies also contributed to the observed variability. The heterogeneity in prompt design was further compounded by the lack of a standardized differential diagnosis list size (*k* value) used for the differential accuracy metric. Our manual review of the methodologies revealed that the k value ranged predominantly from *k* = 3 (observed in 14 studies) to *k* = 5 (observed in 4 studies). This non-uniformity limits the comparability of effect sizes across studies. Such variability highlights the critical need for researchers to adopt standardized evaluation protocols, such as a universal reporting of Hit@3 or Hit@5, in future investigations to ensure the reliability and comparability of pooled estimates. The datasets utilized for each study also varied in source, complexity, and image quality, which may have influenced GPT performance. One of the most prominent limitations was the lack of standardized comparator benchmarks across the studies. Some studies defined ground truth as the written diagnosis in online resources, while others defined ground truth as the listed diagnosis by an attending radiologist in a radiological report. Without such benchmarking, it remains unclear how these models perform relative to current standards of care. Additionally, this study only focuses on GPT, a single LLM.

Though limiting our study to GPT models may strengthen our observations of these specific models, its generalizability to the myriad LLMs used in medical tasks is unclear. Future research may perform similar analyses of LLMs such as Google's Gemini and Anthropic's Claude to assess model-specific strengths and weaknesses. The recently released version of GPT (GPT-5) was also not included in this analysis, as its release date was after the study's cutoff date. The addition of such analyses to the literature may also elucidate larger trends regarding LLM performance in this domain. In our GLMM, we focused on variables that were most consistently reported across the included studies, such as model version, input modality, history provision, and study year. However, future studies should aim to incorporate additional factors, including imaging modality and body region, and provide stratified accuracy estimates, as these variables may significantly influence GPT performance.

In summary, while our findings underscore the considerable potential of advanced GPT models to support radiologic diagnostics, they also highlight critical areas requiring improvement, including modality-specific training, clinical context integration, and fine-tuning using targeted datasets. Addressing these aspects through focused research and methodological refinements will be essential in moving toward the effective clinical integration of LLMs in radiology.

## Data Availability

The raw data supporting the conclusions of this article will be made available by the authors, without undue reservation.

## Supplementary material

SUPPLEMENTARY FIGURE S1 Bias assessment of the selected papers using the NOS tool. NOS was used to assess the risk of bias for all the studies selected in this systematic review and meta-analysis. Two reviewers evaluated each study in the meta-analysis independently across three NOS domains: Selection, Comparability, and Outcome. Scores were summed to yield an overall bias rating, with higher scores indicating lower risk of bias. Studies were categorized as low, moderate, or high risk of bias based on their total NOS scores. This assessment informed the interpretation of pooled diagnostic accuracy and study heterogeneity.
