# Supplementary figures and images for: A systematic review and meta-analysis of GPT-based differential diagnostic accuracy in radiological cases: 2023–2025
Source: Front Radiol. 2025 Oct 28;5:1670517. doi: 10.3389/fradi.2025.1670517 (PMC12602482; doi:10.3389/fradi.2025.1670517)

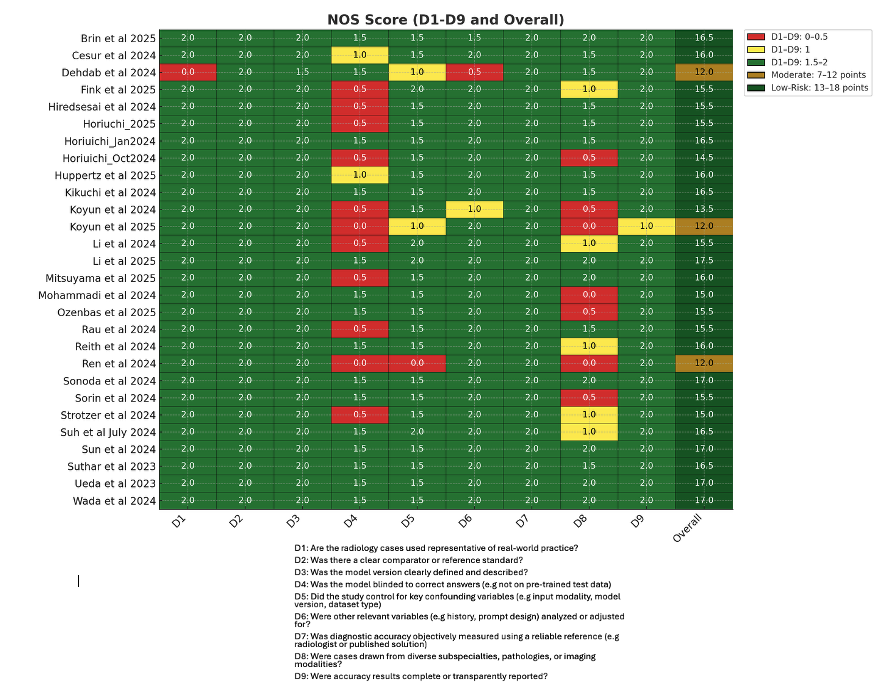

Supplement: SUPPLEMENTARY FIGURE S1 — Bias assessment of the selected papers using the NOS tool. NOS was used to assess the risk of bias for all the studies selected in this systematic review and meta-analysis. Two reviewers evaluated each study in the meta-analysis independently across three NOS domains: Selection, Comparability, and Outcome. Scores were summed to yield an overall bias rating, with higher scores indicating lower risk of bias. Studies were categorized as low, moderate, or high risk of bias based on their total NOS scores. This assessment informed the interpretation of pooled diagnostic accuracy and study heterogeneity. [file Image1.png]
