# Supplementary material for: A systematic review and meta-analysis of GPT-based differential diagnostic accuracy in radiological cases: 2023–2025
Source: Front Radiol. 2025 Oct 28;5:1670517. doi: 10.3389/fradi.2025.1670517 (PMC12602482; doi:10.3389/fradi.2025.1670517)
Supplement: Supplementary file 2 [file Table1.docx]

**Supplementary Table 1: Comparison of Access Date versus Differential Accuracy Performance of GPT Models.** This table examines the relationship between the access date of studies and each GPT model’s performance by extracting the publication year (2023, 2024, 2025). Performance is evaluated using differential accuracy, calculated separately for each year. The results are tabulated to highlight trends in GPT’s performance over time. Results were collapsed to the study level by summing correct predictions and total images per study. For each subgroup, we report the Study-level median accuracy and the interquartile range (IQR; Q3–Q1)**.**

| Model Pub. Year | Total Conditions | Accuracy [95% C.I] | Study-level Median Accuracy (IQR) |
| --- | --- | --- | --- |
| GPT-3.5 |  |  |  |
| 2023 | NA | NA | NA |
| 2024 | 1565 | 38.77% [36.30, 41.14] | 37.01% (13.16) |
| 2025 | 115 | 26.10% [18.06, 34.11] | 26.18% (0.00) |
| GPT-4 |  |  |  |
| 2023 | 534 | 57.49% [53.29, 61.68] | 59.01% (1.15) |
| 2024 | 1863 | 54.12% [51.83, 56.36] | 58.50% (10.62) |
| 2025 | 265 | 70.91% [65.47, 76.40] | 67.42% (26.61) |
| GPT-4T |  |  |  |
| 2023 | NA | NA | NA |
| 2024 | 751 | 66.97% [63.13, 70.34] | 70.60% (0.00) |
| 2025 | 50 | 94.00% [87.74, 100.00] | 94.00% (0.00) |
| GPT-4V |  |  |  |
| 2023 | NA | NA | NA |
| 2024 | 1610 | 46.70% [44.27, 49.15] | 46.19% (40.30) |
| 2025 | 696 | 32.22% [28.72, 35.76] | 33.05% (2.18) |
| GPT-4o |  |  |  |
| 2023 | NA | NA | NA |
| 2024 | 545 | 56.69% [52.53, 60.81] | 51.83% (16.84) |
| 2025 | 858 | 57.57% [54.26, 60.88] | 56.83% (11.17) |
