# Supplementary material for: A systematic review and meta-analysis of GPT-based differential diagnostic accuracy in radiological cases: 2023–2025
Source: Front Radiol. 2025 Oct 28;5:1670517. doi: 10.3389/fradi.2025.1670517 (PMC12602482; doi:10.3389/fradi.2025.1670517)
Supplement: Supplementary file 3 [file Table2.docx]

**Table S2. Generalized Linear Mixed-Effects Model (GLMM) Results of GPT Differential Accuracy Across Key Variables Excluding Moderate Risk of Bias Studies.**

Results of the generalized linear mixed-effects model (binomial, random intercept by study) evaluating predictors of ChatGPT differential diagnostic accuracy across all included studies except moderate risk of bias studies (n = 3). Odds ratios greater than 1 indicate improved accuracy, while values less than 1 indicate reduced accuracy. Significant predictors included ChatGPT model type, modality (text vs visual), history provision, and data source. The fixed effects are the predictors, whereas the random effect is the study-level intercept, which accounts for clustering of multiple observations within the same article.

| Predictor Variable | Odds Ratio (SE) | 95% CI | P Value |
| --- | --- | --- | --- |
| Differential Diagnosis Accuracy |  |  |  |
| GPT-4T/4V/4/4o (vs. GPT-3.5) | 1.84 | (1.61, 2.12) | <.**001** |
| Visual Analysis (vs. Textual Analysis) | 0.27 | (0.18, 0.42) | **<.001** |
| Provided History: Yes (vs. No) | 1.28 | (1.11, 1.49) | **<.001** |
| Data Acquisition: Public (vs. Private) | 0.38 | (0.17, 0.82) | **.014** |
| Year (continuous, 2023-2025) | 1.23 | (0.66, 2.33) | .516 |
